# Studying the Function of Phytoplasma Effector Proteins Using a Chemical-Inducible Expression System in Transgenic Plants

**DOI:** 10.3390/ijms222413582

**Published:** 2021-12-18

**Authors:** Keziah M. Omenge, Florian Rümpler, Subha Suvetha Kathalingam, Alexandra C. U. Furch, Günter Theißen

**Affiliations:** 1 Department of Genetics, Matthias Schleiden Institute for Genetics, Bioinformatics and Molecular Botany, Friedrich Schiller University Jena, Philosophenweg 12, 07743 Jena, Germany; keziah.moraa.omenge@uni-jena.de (K.M.O.); florian.ruempler@uni-jena.de (F.R.); subhasuvetha.kathalingam@uni-jena.de (S.S.K.); 2 Department of Plant Physiology, Matthias Schleiden Institute for Genetics, Bioinformatics and Molecular Botany, Friedrich Schiller University Jena, Dornburger Str. 159, 07743 Jena, Germany; Alexandra.Furch@uni-jena.de

**Keywords:** *Arabidopsis thaliana*, effector protein, inducible gene expression, MADS-box gene, phytoplasma, transcription factor, virulence factor

## Abstract

Phytoplasmas are bacterial pathogens that live mainly in the phloem of their plant hosts. They dramatically manipulate plant development by secreting effector proteins that target developmental proteins of their hosts. Traditionally, the effects of individual effector proteins have been studied by ectopic overexpression using strong, ubiquitously active promoters in transgenic model plants. However, the impact of phytoplasma infection on the host plants depends on the intensity and timing of infection with respect to the developmental stage of the host. To facilitate investigations addressing the timing of effector protein activity, we have established chemical-inducible expression systems for the three most well-characterized phytoplasma effector proteins, SECRETED ASTER YELLOWS WITCHES’ BROOM PROTEIN 11 (SAP11), SAP54 and TENGU in transgenic *Arabidopsis thaliana*. We induced gene expression either continuously, or at germination stage, seedling stage, or flowering stage. mRNA expression was determined by quantitative reverse transcription PCR, protein accumulation by confocal laser scanning microscopy of GFP fusion proteins. Our data reveal tight regulation of effector gene expression and strong upregulation after induction. Phenotypic analyses showed differences in disease phenotypes depending on the timing of induction. Comparative phenotype analysis revealed so far unreported similarities in disease phenotypes, with all three effector proteins interfering with flower development and shoot branching, indicating a surprising functional redundancy of SAP54, SAP11 and TENGU. However, subtle but mechanistically important differences were also observed, especially affecting the branching pattern of the plants.

## 1. Introduction

Phytoplasmas are obligate bacterial pathogens of both plants and insects [1]. In nature, phytoplasmas are spread and introduced into the tissue of new host plants by insect vectors feeding on noninfected plants [1]. Typical insect vectors of phytoplasmas are leafhoppers, planthoppers and cicadas, all belonging to the insect order Hemiptera [2]. As with any tritrophic relationship, the dynamic of a phytoplasma outbreak and the spread from plant to plant depends on the spatiotemporal overlap of vulnerable host plants, pathogens, and vectors, each being influenced by many biotic and abiotic factors [3,4].

After plant infection, phytoplasmas live in the phloem, and cause a plethora of morphological changes to the host plant, including bolting, witches’ broom, dwarfism, reddening or yellowing of leaves and stems, virescence, phyllody, and phloem necrosis [1]. At least some of these symptoms are induced by the secretion of virulence factors, termed effector proteins, into the cytoplasm of the host plant sieve elements from where they are distributed systemically through the plant [1,5,6]. One of the well-characterized phytoplasma effector proteins, SECRETED ASTER YELLOWS WITCHES’ BROOM PROTEIN 54 (SAP54) from *Candidatus* Phytoplasma asteris strain Aster Yellows Witches’ Broom (AY-WB), has been shown to cause phyllody, i.e., the development of vegetative leaf-like structures instead of floral organs [7]. SAP54 exerts at least some of its effects by the degradation of specific MADS-domain transcription factors (MTFs) via the ubiquitin/26S proteasome pathway of the host plant, with the help of some variants of the shuttle protein RADIATION SENSITIVE 23 (RAD23) [8,9,10]. Some of the targeted MTFs specify floral organ identity, hence the depletion of these MTFs ultimately leads to homeosis, i.e., changes in organ identity [9].

Another effector protein, SAP11, has been reported to induce shoot proliferation and leaf shape changes of plants due to the destabilization of TCP transcription factors, particularly the class II TCPs [11,12,13]. TCPs are a family of plant-specific transcription factors that control many plant developmental processes, with class II TCPs mainly being involved in leaf development, shoot branching and flavonoid biosynthesis [14,15].

TENGU, a solely 38-amino acid effector protein secreted by phytoplasma, was first identified from Ca. P. asteris strain Onion Yellows (OY) [16]. It has been observed that *TENGU* induces dwarfism and witches’ broom symptoms when expressed in *Arabidopsis* *thaliana* (henceforth Arabidopsis) and *Nicotiana benthamiana* plants [16], and that it causes plant sterility by interfering with the jasmonic acid and auxin dependent pathways of flower development [17]. Although direct interaction partners of TENGU have not yet been identified, it has been shown that plant host factors with protease activity are required for the processing of TENGU in order to generate functional peptides [18].

The genes encoding SAP54, SAP11 and TENGU each belong to a family of highly conserved homologs that can be found in diverse (but not necessarily all) phytoplasma strains, and that are likely not only transferred vertically, but also horizontally [18,19,20,21].

Phytoplasma strains usually secrete complex mixtures of effector proteins into the phloem of their host plants, comprising more than the three proteins mentioned above, which makes it difficult to determine the contribution of individual factors to phenotypic effects in infected plants [5,7]. Therefore, in a number of studies, individual effector proteins have been expressed in transgenic, but non-infected plants [7,9,11,16]. Remarkably, expression of SAP54, SAP11 and TENGU in Arabidopsis brings about already almost the complete syndrome of phenotypic changes known from phytoplasma infections [7,11,16], suggesting significant functional redundancy of effector proteins.

Despite the general effects outlined above, specific phenotypic consequences of phytoplasma effector proteins may depend on the developmental stage of the host, such as which kinds of meristems (vegetative, floral) exist, and at what developmental stage they are, when effector proteins arrive [22]. However, in all transgenic studies of *SAP54*, *SAP11* or *TENGU* reported so far, the cDNAs of the genes were expressed under the control of constitutive promotors such as the Cauliflower Mosaic Virus (CaMV) 35S promotor or the maize ubiquitin 1 promotor (e.g., [7,9,11,13,16,20]). Consequently, effector gene expression already initiated at germination stage, and the gene products remained present throughout all plant developmental stages. This expression pattern, however, is far from what occurs in nature, where the time point of phytoplasma infection as well as phytoplasma proliferation dynamics define effector protein titers at different developmental stages of the host plant.

This study aims to establish inducible expression systems for the three phytoplasma effector proteins SAP54, SAP11 and TENGU, and to investigate the relevance of the timing of effector protein expression. We generated transgenic plants with DNA constructs for 17-β-estradiol (henceforth β-estradiol) inducible expression of *SAP54*, *SAP11* and *TENGU*, utilizing a previously established chimeric transcription factor system [23]. The system enables the expression of the different phytoplasma effector proteins during different periods of Arabidopsis development (from seedling to flowering stage). Quantitative reverse transcription PCR (qRT-PCR) and confocal laser scanning microscopy (CLSM) demonstrated dynamic effector protein expression upon induction. Detailed phenotypic analysis showed differences in disease phenotype strength and manifestation depending on when expression was induced. They also revealed subtle differences in effector protein effects not reported before, such as *SAP11* and *TENGU* affecting different branch orders during induction of shoot proliferation.

The further potential of stage-specific expression of phytoplasma effector proteins in transgenic plants for future investigations is indicated.

## 2. Results

### 2.1. Establishment of an Inducible Expression System for Phytoplasma Effector Proteins

**Figure 1:**
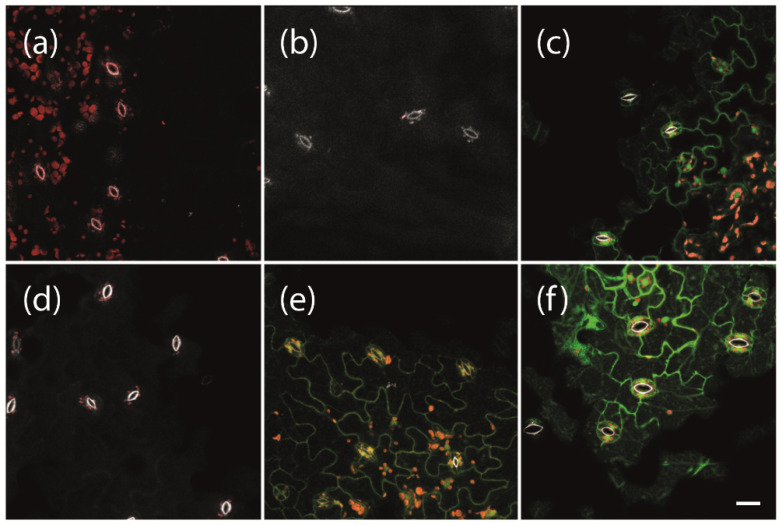
Induction of GFP expression depending on β-estradiol concentrations. Two-week-old (**a**) non-transgenic Arabidopsis Col-0 wild type plants, (**b**) transgenic plants carrying pBb7m34GW-35S::XVE vector with no coding sequence cloned under control of the LexA promoter element (LexA), (**c**) 35S::mGFP6, and (**d**–**f**) LexA::mGFP6 plants grown on non-inductive medium (NM) were either (**d**) kept on NM or transferred to inductive medium (IM) with β-estradiol concentrations of (**e**) 2 or (**f**) 5 µM, respectively. After 24 h, fluorescence signals were recorded. Whereas wild type, LexA and LexA::mGFP6 plants that were kept on NM showed no GFP signals, GFP fluorescence signals were observed in the leaves of 35S::mGFP6 and LexA::mGFP6 plants grown on IM with 2 and 5 µM β-estradiol, respectively. Pictures were taken at X400 magnification at an excitation wavelength of 488 nm and emission spectra were recorded for GFP fluorescence (green), chlorophyll autofluorescence (red) and the autofluorescence of strengthened cell walls of stomata (white), separately. Scale bar corresponds to 100 µm.

To enable the expression of phytoplasma effector proteins for a specific duration of interest at a given developmental stage of the experimental host plant Arabidopsis, we used a previously established system for β-estradiol inducible protein expression [23]. We first tested which concentrations of β-estradiol are suitable under our conditions to induce strong gene expression without damaging the plants. We used transgenic plants carrying a LexA::mGFP6 construct for β-estradiol inducible expression of the mutated green fluorescent protein mGFP6. Plants were grown on non-inductive medium (NM) for 2 weeks and subsequently transferred to inductive medium (IM) containing 2, 5, or 10 µM β-estradiol, respectively. After 24 h of induction, leaves were screened for GFP signals by CLSM (Figure 1). Neither wild type plants, transgenic plants carrying the pBb7m34GW-35S::XVE vector with no coding sequence cloned under control of the LexA operator (termed ‘LexA’ henceforth for simplicity), nor transgenic LexA::mGFP6 plants kept on NM, showed any green fluorescence signal (Figure 1a,b,d). In contrast, LexA::mGFP6 plants showed clear GFP signals in the cytoplasm of the epidermis cells with a typical puzzle-like shape after induction with 2 or 5 μM β-estradiol for 24 h (Figure 1e,f). The signal at 5 μM appears to be even stronger than that generated by the CaMV-35S promoter (Figure 1c), revealing that quite a strong gene expression could be achieved under our conditions. Plants treated with 10 μM β-estradiol showed yellowing of leaves and signs of withering after 24 h of induction. Hence, all further inductions were conducted at a β-estradiol concentration of 5 μM.

### 2.2. Comparative Time-Course Analyses of SAP54, SAP11, and TENGU Induction

**Figure 2:**
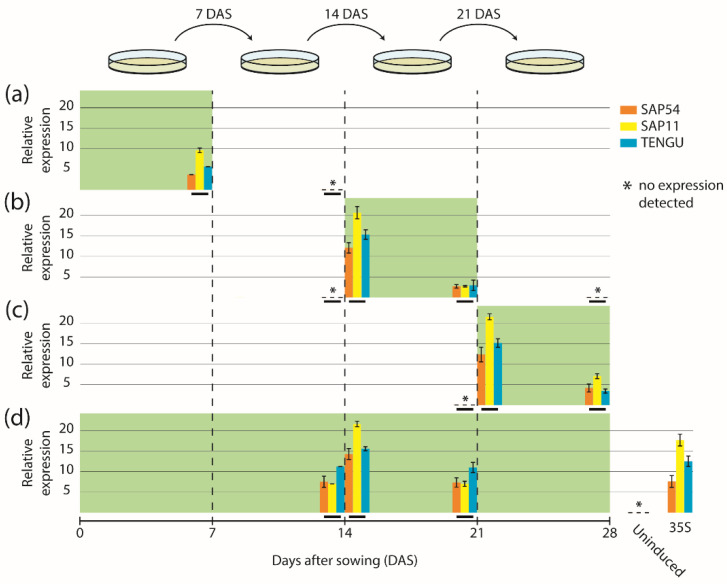
Expression time-course of mGFP6-SAP54, mGFP6-SAP11 and mGFP6-TENGU mRNAs. Arabidopsis LexA::mGFP6-SAP54, LexA::mGFP6-SAP11, and LexA::mGFP6-TENGU plants were grown following four different induction regimes—(**a**) induction at germination stage, (**b**) induction at seedling stage, (**c**) induction at flowering stage, and (**d**) continuous induction. Green background depicts incubation on IM. Transcript levels of *mGFP6-SAP54, mGFP6-SAP11*, and *mGFP6-TENGU* in leaves were measured at different time points and expressed relative to the geometric mean of the expression of the housekeeping genes *ADENOSINE PHOSPHORIBOSYL TRANSFERASE 1* (*APT1*) and *ACTIN 2* (*ACT2*). Relative expression levels of *mGFP6-SAP54, mGFP6-SAP11*, and *mGFP6-TENGU* are shown as orange, yellow and blue bar plots, respectively, placed on a timescale according to the time point of sampling (highlighted by horizontal black bars). (**a**) Plants induced at germination stage were sampled after 7 d on IM, and 7 d after the plant was transferred back to NM. (**b**) Plants induced at seedling stage were sampled immediately prior to the transfer to IM, 24 h after subjecting the plant to IM, after 7 d on IM, and 7 d after the plant was transferred back to NM. (**c**) Plants induced at flowering stage were sampled immediately prior to the transfer to IM, 24 h after subjecting the plant to IM, and after 7 d on IM. (**d**) Continuously induced plants were sampled immediately prior to the transfer to a new plate of IM 14 d after sowing (DAS), 24 h after subjecting the plants to new IM and immediately prior to the transfer to a new plate of IM 21 DAS. In panel (**d**) additionally the measured transcript levels of LexA::mGFP6-SAP54, LexA::mGFP6-SAP11, and LexA::mGFP6-TENGU plants grown on NM throughout (uninduced control) and plants expressing the mGFP6-effector genes fusions under control of the CaMV-35S promoter are shown.

We next sought to determine the expression dynamics of the fusion constructs mGFP6-SAP54, mGFP6-SAP11, and mGFP6-TENGU in Arabidopsis leaves over time. We induced expression following four different induction regimes (induction at germination stage, seedling stage, flowering stage, and continuous induction) and analyzed effector gene transcript levels in leaves by qRT-PCR at different time points (Figure 2). Seven d after LexA::mGFP6-SAP54, LexA::mGFP6-SAP11, and LexA::mGFP6-TENGU seeds had been sown on IM, seedlings showed moderate effector gene expression, whereas no effector gene transcripts were detected 7 d after the end of the induction period (Figure 2a). If expression was induced at seedling and at flowering stage, high effector gene transcript levels were detected 24 h after subjecting the plants to IM (Figure 2b,c), with transcript levels exceeding that of plants expressing the effector genes under control of the CaMV-35S promoter. Transcript levels reduced remarkably after seven days on IM. However, as demonstrated by plants subjected to continuous induction, transfer to a new plate of IM restored high transcript levels within 24 h after transfer (Figure 2d). It therefore appears likely that β-estradiol degradation in the medium is causal for the transcript level reduction during the induction phase. We did not observe any effector gene transcripts in leaves of plants that had not been induced by β-estradiol, indicating that the inducible system does not cause unwanted background expression in the absence of the chemical inducer.

**Figure 3:**
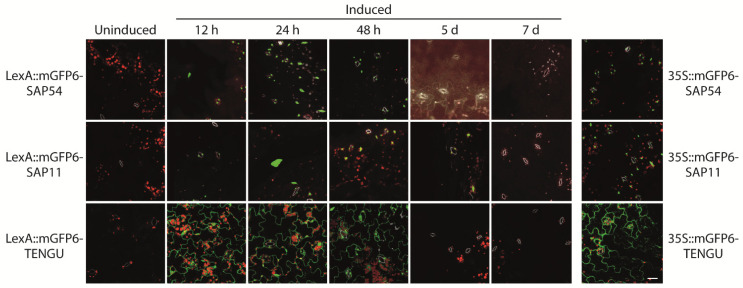
Time course of mGFP6-SAP54, mGFP6-SAP11, and mGFP6-TENGU fusion protein expression after induction. 2-week-old Arabidopsis LexA::mGFP6-SAP54, LexA::mGFP6-SAP11, and LexA::mGFP6-TENGU plants grown on NM were transferred to IM and fluorescence signals were recorded after 12 h, 24 h, 48 h, 5 d, and 7 d. For comparison fluorescence signals of plants constitutively expressing mGFP6-SAP54, mGFP6-SAP11 and mGFP6-TENGU, respectively under control of the CaMV-35S promotor are shown. Pictures were taken at X400 magnification at an excitation wavelength of 488 nm and emission spectra were recorded for GFP fluorescence (green), chlorophyll autofluorescence (red) and autofluorescence of strengthened cell walls of stomata (white) separately. Scale bar corresponds to 100 µm.

LexA::mGFP6-SAP54, LexA::mGFP6-SAP11, and LexA::mGFP6-TENGU transgenic plants induced at seedling stage were screened for GFP signals in leaves after 12 h, 24 h, 48 h, 5 and 7 d cultivation on IM. Strong GFP fluorescence was detected after 12, 24 and 48 h of induction for all three effector proteins (Figure 3). The fluorescence intensity was comparable to that observed for plants continuously expressing mGFP6-effector protein fusions under control of CaMV-35S. Whereas LexA::mGFP6-TENGU produced strong GFP signals in the cytoplasm of epidermis cells, LexA::mGFP6-SAP54 and LexA::mGFP6-SAP11 plants showed isolated spots of GFP fluorescence suggesting nuclear localization. Fluorescence signals considerably weakened after 5 d, and no GFP signals were detectable after 7 d of cultivation on IM, very likely due to β-estradiol degradation in the medium (Figure 3). LexA::mGFP6-SAP54, LexA::mGFP6-SAP11, and LexA::mGFP6-TENGU plants germinated and cultured on NM throughout showed no GFP signals.

### 2.3. Timely Induction of Effector Proteins Affects Plant Habit of Arabidopsis

**Figure 4:**
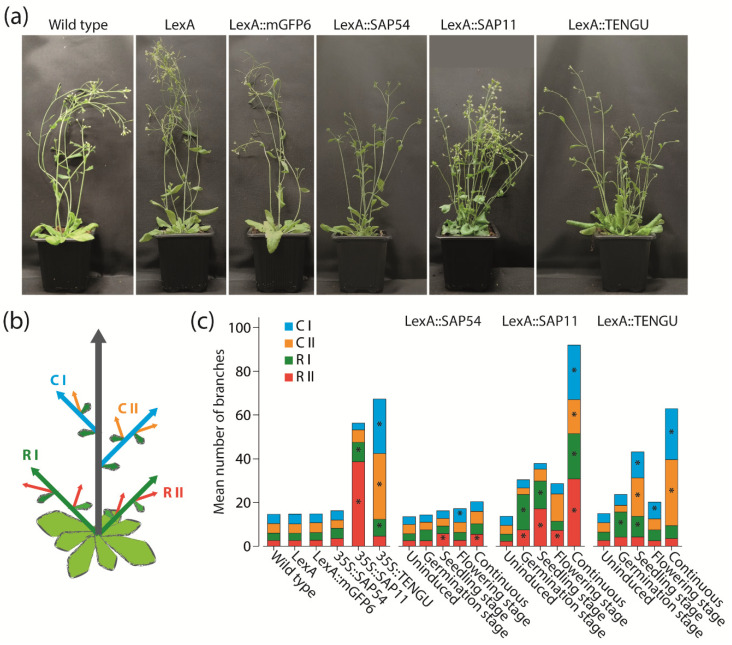
Branching phenotype observed in Arabidopsis upon SAP54, SAP11, and TENGU expression. (**a**) Plant habit of non-transgenic Arabidopsis Col-0 wild type plants, transgenic plants carrying pBb7m34GW-35S::XVE vector with no coding sequence cloned under control of the LexA promoter element (LexA), LexA::mGFP6, LexA::SAP54, LexA::SAP11, and LexA::TENGU plants that were grown on IM for 28 d (continuous induction). (**b**) Scheme illustrating shoot branch orders of Arabidopsis with the main shoot (grey arrow), primary rosette branches (R I, green arrows), secondary rosette branches (R II, red arrows), primary cauline branches (C I, blue arrows), and secondary cauline branches (C II, orange arrows). (**c**) Number of primary and secondary rosette and cauline branches of effector protein expressing plants and control lines counted 28 DAS. LexA::SAP54, LexA::SAP11, and LexA::TENGU plants were either grown on NM throughout (uninduced), or induced following the induction regimes illustrated in Figure 2. Arabidopsis wild type plants, LexA, LexA::mGFP6, 35S::SAP54, 35S::SAP11, and 35S::TENGU plants were grown on IM throughout. Branch numbers significantly differing from wild type values are indicated by an asterisk (Kruskal–Wallis test followed by pairwise Mann–Whitney U tests, Bonferroni corrected, * *p* ≤ 0.05, *n* = 10–20). As can be seen in the plant habit images in panel (**a**), some effector protein expressing plants additionally displayed rosette leaf alterations, such as leaf roll and curly/crinkled leaves.

Because SAP11 and TENGU are known to induce shoot proliferation and witches’ broom symptoms [11,16], we aimed to investigate the impact of expression time of SAP54, SAP11, and TENGU on the branching of Arabidopsis. Consistent with the literature, SAP11 and TENGU were able to induce shoot proliferation (Figure 4). The increase in the number of shoot branches was highest under continuous effector protein expression, with a total number of shoot branches similar or higher to that observed in 35S::SAP11 and 35S::TENGU plants, respectively (Figure 4c). Induction of SAP11 and TENGU expression at either the germination, seedling or flowering stage caused shoot proliferation as well, although with a less drastic increase in shoot branch number.

Interestingly, a direct comparison of 35S::SAP11 and 35S::TENGU plants revealed that both effector proteins interfere with different orders of shoot branches. Whereas plants overexpressing SAP11 show the highest increase in primary and secondary rosette branch numbers, the witches’ broom phenotype caused by the overexpression of TENGU is mainly brought about by an increased number of primary and secondary cauline branches (Figure 4c). The same tendency for an increase in shoot number of certain branch orders was observed for LexA::SAP11 and LexA::TENGU plants, albeit continuously induced LexA::SAP11 plants showed heavily increased shoot numbers of all branch orders.

In previous studies where SAP54 has been expressed in Arabidopsis under control of the CaMV-35S promoter, no effect of SAP54 expression on shoot branching has been described [7]. Surprisingly, LexA::SAP54 plants induced at the seedling or flowering stage, as well as continuously induced LexA::SAP54 plants, showed slightly increased numbers of first order cauline branches and secondary order rosette branches, whereas LexA::mGFP6 control plants grown under the same conditions did not (Figure 4c).

### 2.4. Timely Induction of Effector Protein Expression Alters Arabidopsis Flower Development

**Figure 5:**
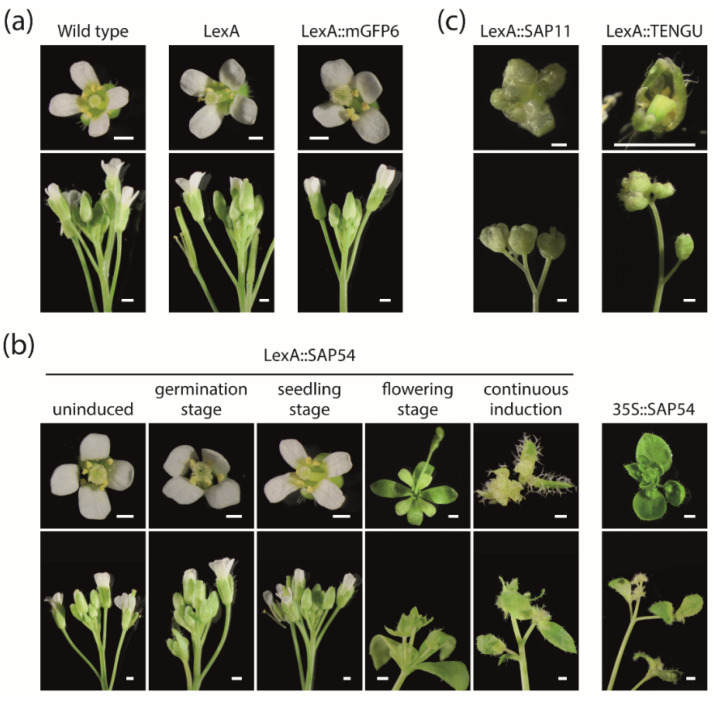
Floral phenotypes observed upon induction of SAP54, SAP11, and TENGU expression in Arabidopsis. (**a**) Flowers (top) and inflorescences (bottom) of Arabidopsis Col-0 wild type plants, LexA empty vector control plants and LexA::mGFP6 plants grown on IM throughout (continuous induction). (**b**) Flowers and inflorescences of LexA::SAP54 plants that were either grown on NM throughout (uninduced), induced at germination, seedling or flowering stage, or grown on IM throughout (continuous induction). Flowers of 35S::SAP54 plants are shown for comparison. (**c**) Flowers and inflorescences of LexA::SAP11 and LexA::TENGU plants grown on IM throughout. All images were recorded 28 DAS. Scalebars correspond to 0.5 mm.

As SAP54 has been reported to cause phyllody, i.e., the development of leaf-like structures instead of floral organs, if expressed in Arabidopsis, we conduct a detailed analysis of plants’ floral phenotypes depending on the onset of effector gene expression. Neither Arabidopsis wild type plants, nor LexA empty vector control plants or LexA::mGFP6 plants displayed floral abnormalities if grown on IM (Figure 5a). Similarly, LexA::SAP54 plants grown on NM throughout, as well as plants induced at germination or seedling stage showed no signs of phyllody (Figure 5b). In contrast, LexA::SAP54 plants induced at the flowering stage as well as continuously induced plants exhibited strong phyllody symptoms including partial or complete transformation of floral organs into leaf-like structures, development of flowers within a flower and the proliferation of undifferentiated cells (Figure 5b). Phyllody symptoms were strongest for continuously induced LexA::SAP54 plants exceeding those observed for 35S::SAP54 plants (Figure 5b).

Interestingly, we also observed abnormal flowers in continuously induced LexA::SAP11 and LexA::TENGU plants (Figure 5c). Two or more flowers grew from a single point on the stem. The flowers and their flower stalks were weak and pale (Figure 5c). The majority of these plants produced sterile flowers. It appears unlikely that these abnormalities are a result of the β-estradiol treatment, as neither wild type plants, nor LexA empty vector control plants, or LexA::mGFP6 plants grown on IM throughout exhibited any notable flower alterations (Figure 5a).

## 3. Discussion

After infection by phytoplasma, host plants frequently develop a drastic and often even devastating disease syndrome, typically including phyllody, virescence, sterility, a plant habit resembling witches’ brooms and dwarfism. Studying the molecular and physiological basis of these symptoms has been hampered by several facts. For example, phytoplasma cells secrete a whole cocktail of effector proteins into the phloem of their host plants, so that it is difficult to attribute the different effects on the phenotype to individual effector proteins. To deal with this drawback, individual effector proteins have been expressed in transgenic model plants, especially Arabidopsis [7,11,16].

Moreover, the phenotypic consequences of effector proteins may depend on plant age and developmental state, and hence the time when during the development of a host plant phytoplasma infection and effector protein secretion occurs. To overcome the experimental limitations of constitutive ectopic overexpression of effector proteins, we have established inducible expression systems for the three most well-characterized effector proteins that act in plants, SAP11, SAP54 and TENGU. Our systems employ the chimeric transcriptional activator XVE and a Gateway-compatible plasmid collection [23,24]. The systems enable the expression of the effector proteins for a specific duration of interest at a given developmental stage of the experimental host plant Arabidopsis.

Induction of gene expression in four different ways (continuous induction, or at germination stage, seedling stage, or flowering stage) led to strong and induction specific accumulation of all three effector proteins fused to a GFP derivative (mGFP6), and hence revealed the versatility of our approach.

As an initial application of our expression systems, we investigated the impact of the expression time of SAP11, SAP54 and TENGU on the branching pattern of Arabidopsis. SAP11 and TENGU are known for their ability to lead to an increase in the number of shoot branches if expressed constitutively [11,16]. The potential of SAP11 and TENGU was also obvious in our experiments, with the increase in the number of shoot branches being highest under continuous effector protein expression. Induction of SAP11 and TENGU expression at germination, seedling or flowering stage also led to an, albeit weaker, increase in shoot branching (Figure 4c).

An effect on shoot branching had not been reported for SAP54, however [7]. It came to us as a surprise, that the induction of SAP54 expression continuously or at seedling or flowering stage also led to a slight increase in the numbers of first order cauline branches and secondary order rosette branches of transgenic Arabidopsis (Figure 4c). Thus, the phenotypic effects of the expression of the different effector proteins might be more similar than was previously known, indicating considerable functional redundancy of the different proteins. Whether this indicates functional importance of these effects remains speculative, however, since “collateral damages” currently cannot be excluded [25]. The phenotypic redundancy is also remarkable given that previous studies indicated that the different effector proteins have considerably different target proteins (TCPs and MADS-domain proteins for SAP11 and SAP54, respectively; unknown for TENGU) [12,13]. Based on the available data, it is tempting to speculate, however, that the targeting of the different effector proteins is more promiscuous than was previously appreciated.

A detailed comparison of plants expressing SAP11 and TENGU showed that the two effector proteins affect different orders of shoot branches (Figure 4). Whereas most induction regimes as well as the overexpression of SAP11 caused the highest increase in primary and secondary rosette branch numbers, TENGU expression mainly causes an increase of primary and secondary cauline branches (Figure 4c). This observation may suggest that the overall expression level of the different phytoplasma proteins depending on, among other things, the promoters being used, determines which branch primordia are affected the most. It could also be that the reason for the differences between SAP11 and TENGU effects are different targets of these proteins. These target proteins might have different activities and be of different functional importance in the different branch primordia. This hypothesis is, however, currently difficult to test, because the targets of TENGU are not yet known.

Of course, the initial experiments outlined here could easily be extended in many different ways. For example, numerous different induction regimes are conceivable. The combined analysis of effector gene expression, protein accumulation and phenotype dynamics could give insights about *in planta* effector protein stability. Furthermore, this would allow for identification of developmental stages at which the plant is especially susceptible to effector protein activity. Another trivial extension would be the study of other effector proteins beyond the three investigated here.

Further investigations may use tissue-specific rather than ubiquitously active promoters. Since phytoplasma cells live in the cytoplasm of sieve elements, the use of phloem-specific promoters would be an obvious choice, but also other options are conceivable. Promoters that drive different expression patterns, including some specific for developing sieve elements and companion cells, companion cells alone and phloem pole pericycle cells, are already available as XVE inducible entry clones of a Gateway-compatible system [24]. Since phytoplasma effector proteins are small and are systemically distributed from the phloem throughout the plant, the usage of such tissue specific promoters would simulate a phytoplasma infection more accurately than ubiquitously active promoters.

Arabidopsis is arguably not the most important host of phytoplasma, at least from an agronomic and economic point of view. Therefore, it may also appear useful to apply our approach to other species, especially crop and horticultural plants that are amenable to transformation technology.

## 4. Materials and Methods

### 4.1. Plant Material and Growth Conditions

All plant lines for inducible and constitutive effector protein expression were generated via floral dip [26] of *Arabidopsis thaliana* Col-0 plants with transgenic *Agrobacterium tumefaciens* GV3101 cells carrying the expression plasmids described below.

Arabidopsis Col-0 wild-type and transgenic seeds were surface-sterilized for 10 min in sodium hypochlorite, washed five times with sterile water, and stratified at 4 °C in the dark for 2 d. The seeds were either plated on Murashige and Skoog (MS) solid medium without additional supplements (non-inductive medium, NM) or on MS solid medium supplied with 17-β-estradiol (Sigma-Aldrich, St. Louis, MO, USA), dissolved in dimethyl sulfoxide (DMSO) with a final concentration of 2, 5, or 10 µM (inductive medium, IM). The seedlings were grown under a 16-h light/8-h dark photoperiod (light intensity 150–200 µmoL/s/m^2^) at day temperatures of 24–26 °C and night temperatures of 20–21 °C. Seedlings were transferred to new plates 7, 14, 21, and 28 d after sowing (DAS). In total, five different induction regimes were used: (1) uninduced control—seeds were sown on NM and seedlings/plants were transferred to new plates of NM every 7 d; (2) induction at germination stage—seeds were sown on IM and seedlings/plants were transferred to NM 7 DAS and subsequently transferred to new plates of NM every 7 d; (3) induction at seedling stage—seeds were sown on NM, seedlings/plants were transferred to new plates of NM 7 DAS, to IM 14 DAS and back to NM 21 DAS; (4) induction at flowering stage—seeds were sown on NM, seedlings/plants were transferred to new plates of NM 7 DAS and 14 DAS and to IM 21 DAS; (5) continuous induction—seeds were sown on IM, and seedlings/plants were transferred to new plates of IM every 7 d.

### 4.2. Generation of Plasmids for Inducible Effector Protein Expression

All plasmids for β-estradiol inducible protein expression were cloned based on the MultiSite Gateway-compatible inducible system developed by [24]. In brief, the inducible system is based on the chimeric protein XVE, which is composed of the DNA-binding domain of the bacterial repressor LexA (X), the transactivation domain of VP16 (V), and the carboxyl region of the human estrogen receptor (E) [23]. XVE is constitutively expressed under control of the CaMV-35S promoter, whereas the phytoplasma effector proteins are expressed under control of a minimal CaMV-35S promoter adjacent to eight copies of the LexA operator sequence (henceforth referred to as LexA promotor element for simplicity). In the presence of β-estradiol, XVE enters the nucleus and binds to the LexA operator sequences, eventually resulting in β-estradiol induced effector protein expression.

The coding sequence of the secreted part of Ca. P. asteris strain AY-WB *SAP54* (CP000061.1, AYWB_224) was synthesized and cloned previously [27]. The complete coding sequences of Ca. P. asteris strain AY-WB *SAP11* (CP000061.1, AYWB_370), and strain OY-M *TENGU* (AB750355.1) were codon-optimized for *Escherichia coli* and synthesized via the GeneArt Gene Synthesis service of Thermo Fisher Scientific (Thermo Fisher Scientific, Waltham, MA, USA). The sequences encoding for the secreted parts of *SAP54*, *SAP11*, and *TENGU* were PCR amplified to add attB1 and attB2 Gateway cloning sites at 3′ and 5′ end, respectively. For inducible GFP expression, the coding sequence of the enhanced GFP gene mGFP6 was PCR amplified from the plasmid pGreenII-35::mGFP6 [28], while adding attB1 and attB2 sites at the 3′ and 5′ end, respectively. The purified PCR products were recombined in a BP Clonase reaction into the Gateway entry plasmid pDONR221 (Thermo Fisher Scientific, Waltham, MA, USA). The resulting entry plasmids pDONR221-SAP54, pDONR221-SAP11, pDONR221-TENGU, and pDONR221-mGFP6 were subsequently recombined in a multisite LR Clonase reaction, along with entry plasmids p1R4-p35S-XVE (carrying the XVE gene under control of the CaMV-35S promoter followed by the LexA promoter element [24]) and p2R3a-nosT (carrying a NOS-terminator element [24]) into the destination vector pBb7m34GW [29] that carries a Basta resistance gene as a selection marker of transgenic plants. The resulting expression vectors were pBb7m34GW-35S::XVE::nosT-LexA::SAP54::nosT, -35S::XVE::nosT-LexA::SAP11::nosT, -35S-XVE::nosT-LexA::TENGU::nosT and -35S::XVE::nosT-LexA::mGFP6::nosT. In essentially the same way, plasmids for inducible expression of the three effector proteins with an N-terminal mGFP6 fusion were generated. For simplicity, Arabidopsis plants transformed with the abovementioned inducible expression vectors were termed Arabidopsis LexA::SAP54, LexA::SAP11, LexA::TENGU, LexA::mGFP6, LexA::mGFP6-SAP54, LexA::mGFP6-SAP11, and LexA::mGFP6-TENGU, respectively. The coding sequences of the transgenes are given in Table S1.

### 4.3. Generation of Constitutive Expression Vectors

The sequences encoding for the secreted part of SAP54, SAP11, and TENGU were PCR amplified to add XbaI and EcoRI restriction sites for subsequent cloning into the multiple cloning site of pGreen0229mod-35S (pGreen0229 carrying the CaMV-35S cassette with an altered multiple cloning site, see Table S2) to generate pGreen0229mod-35S::SAP54, pGreen0229mod-35S::SAP11 and pGreen0229mod-35S::TENGU. For constitutive expression of mGFP6 the coding sequence of mGFP6 was PCR amplified to add HindIII and XbaI restriction sites for subsequent cloning into pGreen0229mod-35S to generate pGreen0229mod-35S::mGFP6. For constitutive expression of mGFP6-tagged effector proteins the abovementioned PCR products of SAP54, SAP11, TENGU, and mGFP6 were digested using XbaI and ligated to produce mGFP6-SAP54, mGFP6-SAP11 and mGFP6-TENGU DNA fragments. The ligation products were subsequently digested with HindIII and EcoRI, and ligated into the multiple cloning site of pGreen0229mod-35S to generate pGreen0229mod-35S::mGFP6-SAP54, pGreen0229mod-35S::mGFP6-SAP11 and pGreen0229mod-35S::mGFP6-TENGU. Arabidopsis plants transformed with the abovementioned constitutive expression vectors were termed Arabidopsis 35S::SAP54, 35S::SAP11, 35S::TENGU, 35S::mGFP6, 35S::mGFP6-SAP54, 35S::mGFP6-SAP11, and 35S::mGFP6-TENGU, respectively.

### 4.4. Gene Expression Analysis via Quantitative RT-PCR

For each of three biological replicates, 10–20 mg leaf samples of ten plants were pooled and used for isolation of total RNA following the protocol of [30]. RNA extracts were DNaseI digested (Thermo Fisher Scientific, Waltham, MA, USA) and the absence of genomic DNA was tested by PCR using primers for amplification of the *ADENOSINE PHOSPHORIBOSYL TRANSFERASE 1* (*APT1*) gene. Moreover, 1 µg of total RNA was used for cDNA synthesis using RevertAid H Minus Reverse Transcriptase (Thermo Fisher Scientific, Waltham, MA, USA). Quantitative RT-PCR was performed in three technical replicates on a Mx3005P qRT-PCR cycler (Agilent Technologies, Santa Clara, CA, USA) using the Maxima SYBR Green/Rox qPCR Master Mix (Thermo Fisher Scientific, Waltham, MA, USA) with 1 µL of 1:3 diluted cDNA and 0.3 µM of primers. The following PCR cycling program was used: 95 °C for 10 min, 40 cycles of 95 °C for 15 s, 60 °C for 30 s and 72 °C for 30 s. Raw data were analyzed using LinRegPCR software [31,32] to determine primer efficiencies and sample C_T_ values. The C_T_ values for triplicate reactions were averaged. Relative expression quantities were expressed as 2^-ΔC^_T_ with ΔC_T_ being calculated by subtracting the mean C_T_ value of the two housekeeping genes *APT1* and *ACTIN 2* (*ACT2*) from the C_T_ value of the respective effector gene [33]. Expression stabilities of *APT1* and *ACT2* in Arabidopsis leaves have been determined previously [34,35]. The primer sequences used for quantitative RT-PCR are given in Table S3.

### 4.5. Morphological Characterization

Arabidopsis Col-0 wild type and transgenic lines were characterized with respect to number of shoot branches and flower morphology. The number of primary and secondary rosette and cauline branches was manually counted 28 DAS. Five flowers per plant were assessed 28 DAS. Photos of flowers were taken with an Olympus SZX16 stereomicroscope (Olympus Coorporation, Tokio, Japan). For pictures of whole plants, plants were transferred to pots with soil 28 DAS, and photos were taken using Sony α350 camera (Sony, Tokyo, Japan) with Sigma DC 18–200 mm lens (Sigma, Kawasaki, Japan). Statistical significance of differences in shoot branch numbers was determined using Mann–Whitney U tests implemented in SPSS (IBM, Armonk, NY, USA).

### 4.6. Confocal Laser Scanning Microscopy

To analyze protein expression, leaves of Arabidopsis plants carrying LexA::mGFP6-SAP54, LexA::mGFP6-SAP11, LexA::mGFP6-TENGU, and LexA::mGFP6 T-DNA were visualized by CLSM. Fourteen-day-old seedlings grown on NM were transferred to IM, and leaves were harvested after 12 h, 24 h, 48 h, 5 d, and 7 d, mounted on slides, and mGFP6 fluorescence was imaged using a Zeiss 880 laser scanning microscope (Zeiss Microscopy GmbH, Jena, Germany) with a 488 nm laser line. Emission wavelength was recorded separately for mGFP6 fluorescence (color-coded in green), chlorophyll autofluorescence (red) and the autofluorescence of strengthened cell walls (white). Images were taken with a 400× magnification (Plan-Apochromat 40×/0.8). Lambda stacks were created using the 32-channel GaAsP detector followed by Linear Unmixing with ZEN software (Zeiss Microscopy GmbH, Jena, Germany). Z-stacks were taken from specific sample areas, and Maximum Intensity Projections were produced with ZEN software.

## Data Availability

The data presented in this study are available in supplementary material.

## Supplementary Materials

The following are available online at https://www.mdpi.com/article/10.3390/ijms222413582/s1.
